# Relationship between placenta malaria and mother to child transmission of HIV infection in pregnant women in South East Nigeria

**DOI:** 10.1186/s12936-020-03171-2

**Published:** 2020-02-27

**Authors:** Ikechukwu I. Mbachu, Samson D. Ejikunle, Frederick Anolue, Chioma N. Mbachu, Ephraim Dike, Eke Ejikem, Chijioke Okeudo

**Affiliations:** 1 Department of Obstetrics and Gynecology, Nnamdi Azikiwe University, Nnewi Campus, Awka, Anambra Nigeria; 2 Department of Obstetrics and Gynecology, Imo State University Teaching Hospital, Orlu, Nigeria; 3 Department of Paediatrics, Nnamdi Azikiwe University Teaching Hospital, Nnewi, Anambra Nigeria; 4 Department of Obstetrics and Gynecology, Abia State University Teaching Hospital, Aba, Nigeria

**Keywords:** Placenta malaria, Mother to child transmission of HIV

## Abstract

**Background:**

This study determined the rate of mother-to-child transmission (MTCT) of HIV among HIV positive women with placenta malaria and factors associated with placenta malaria.

**Methods:**

This was a prospective observational study of booked HIV positive pregnant women in labour. A smear for malaria parasite was made from blood taken from the placental tissue post-delivery. The baby HIV testing was done with DNA polymerase chain reaction at 6 weeks postpartum. Data on age, parity, gestational age, religion, address, highest educational attainment and knowledge about malaria prevention in pregnancy was obtained with questionnaires and analysed using SPSS version 20. The P-value was set at 0.05 providing a confidence interval of 95%.

**Results:**

A total of 174 booked HIV women participated in this study. The placental malaria parasitaemia prevalence was 44.8%. Overall rate of MTCT of HIV infection was 17.2%. Number of infants with HIV infection among women with maternal placental malarial parasitaemia was 30/78 (38.5%), while it was 0/96 (0%) for women without placenta malaria. There was significant relationship between placenta malaria density and infant HIV status (P-value = 0.001). The relative risk for MTCT of HIV for women with placenta malaria Density > 5000 was 25% with 95% confidence interval of 11.41–54.76%.

**Conclusion:**

The mother-to-child transmission rate of HIV was high among HIV positive women with placental malaria parasitaemia. There is the need to review the malarial treatment and prophylactic measures at least in this group of women and to establish the nature of relationship between placenta malaria and MTCT of HIV infection.

## Background

It is estimated that malaria and HIV infection directly or indirectly contribute to more than 4 million deaths per year [1]. In Africa HIV/AIDS is more prevalent in woman (60%) [2–4]. This translates into high prevalence of vertical transmission of HIV infection in Africa [2, 3]. Mother-to-child transmission (MTCT) is by far the largest source of human immune deficiency virus (HIV) infection in children below the age of 15 year [5].

Malaria during pregnancy in sub-Saharan Africa, affects an estimated 24 million pregnant women; malaria prevalence, may exceed 50% among primigravid and secundigravid woman in malaria-endemic areas [6] and this has generated concern about the potential interactions with HIV infection, especially in sub-Saharan Africa [7–9]. There is evidence that HIV infection appears to impair malarial immunity among pregnant women, as pregnant women infected with HIV demonstrate more frequent and higher density parasitemia than pregnant women not infected with HIV [7–10]. Other studies have also documented faster HIV disease progression and higher viral load among pregnant women with malaria infection. Several studies have documented the fetal complications of placental malaria parasitemia and the association between maternal HIV status and fetal outcome [11–15].

However, only few studies have explored potential role of malaria on MTCT of HIV infection especially in sub-Saharan Africa, where malaria and HIV are endemic. The observations from the few studies showed conflicting findings [16–22]. Ayisi et al. [20] in a study on maternal malaria and perinatal HIV transmission in Western Kenya noted that high density of placenta malaria is associated with increased risk of placenta malaria. However, Briand et al. [23] stated the need for further research on the impact of malaria on the clinical course of HIV and MTCT and antimalaria-antiretroviral interactions based on the conflicting reports by different researchers. Alemu also submitted that more studies will be needed to establish a clear relationship between malaria and MTCT of HIV infection [16]. The World Health Organization (WHO), in a recent paper on malaria and HIV, noted inconsistent results from studies assessing the impact of placenta malaria on MTCT of HIV infection and is calling for more research to shade more light on the impact of malaria on the natural history of HIV and potential therapeutic interactions. This study was carried out to determine the relationship between malaria placenta parasitaemia and MTCT of HIV in a tertiary health centre in Nigeria. It also evaluated the effect of prophylactic and therapeutic treatment of malaria on the placenta malaria density.

## Methods

This was a prospective observational study among all booked HIV positive pregnant women in labour and their infants. This study was conducted over a 5-month period. This study was carried out in the labour ward of the Federal Medical Centre, Imo State, South-East of Nigeria. From December 2016 to March 2017. The centre offers free HIV voluntary counseling and testing, services as well as treatment, care and support of HIV positive patients. In addition, there is a modern laboratory facility equipped for appropriate diagnosis of endemic diseases and laboratory scientists trained in a current malarial diagnosis. The study was conducted among booked HIV positive pregnant women who presented to the labour ward and their infants.

### Inclusion criteria

This included all booked HIV positive pregnant women in labour who consented to the study. The study excluded pregnant women who withheld consent or had major life-threatening opportunistic infection, pregnant women who presented in the third stage of labour and pregnant women who presented in labour but whose initial HIV screening was done outside the study facility.

### Sample size determination

The minimum sample size needed to achieve a precision of 5% at 95% confidence level was determined [24] using the prevalence rate of placenta parasitaemia of 11.3% as documented by a study Inyang-Etoh et al. in Nigeria [25], to a minimum sample size of 170. A total of 174 subjects were recruited and participated in the study. Patients who met the inclusion criteria and gave consent were recruited in the labour ward until the number of the sample was reached. The recruitment was from Monday to Sunday every week for the period of the study.

### Measure of outcome

The main measure of outcome was the prevalence of placenta malaria and rate of mother to child transmission of HIV infection. The secondary outcome measures included relative risk of MTCT of HIV in women who had placenta malaria and factors significantly associated with presence of placenta malaria.

### Study procedure/methods

Explanatory variables included age, parity, gestational age at booking, ethnicity, religion, educational status, occupation, use of IPT (sulfadoxine-pyrimethamine combination) for malaria in pregnancy, sleeping under insecticide-treated nets, use of HAART, CD4 counts, Episiotomy/perinatal tear, duration of rupture of membranes. The women are usually counseled after diagnosis in antepartum period for need for retesting in labour according to the national guideline of Nigeria on PMTCT of HIV. The counseling was reinforced by the researchers and assistants when they present in labour. Relevant information was entered into the question from the notes and any additional information was obtained from the patient on admission in labour ward. The parturient who fulfilled the inclusion criteria were recruited in labour ward. A standard obstetric care for HIV positive women in labour was strictly observed. A smear was made from the placental tissue post-delivery. The newborns were given prophylactic Nevirapine for 6 weeks. The infants were seen in 6 weeks postpartum for infant HIV testing using DNA PCR by collecting the dried blood sample (DBS).

### Placental blood aspiration techniques

As used in the study by Sowunmi et al. [26] the fetal surface of the placenta was positioned upwards. A 21-gauge needle attached to a 5 ml syringe was inserted through the whole thickness of the placental at its thickest point to measure its thickness. The needle was then withdrawn and the approximate thickness of the placental was determined. The needle was re-inserted at the same point such that its tip was placed at a level approximately below half the depth of the estimated placental thickness. The tip of the needle at this position was located in the maternal half of the placenta [27].

After withdrawal, the attached needle was carefully discarded into a sharp object box. Thick blood smear was prepared from placental aspirates and air dried. The smears were stained with freshly prepared 20% Giemsa maintained at a pH of 7.2 for 15 min. The stain was rinsed off in buffered water and left to dry propped diagonally.

### Assessment of quality of blood smears and parasitaemia quantification

The blood smears were examined by light microscopy at low (×10) and high (×100 oil immersion) magnifications. The following parameters were assessed in the stained blood smears obtained by the technique: the count of asexual and sexual forms of *Plasmodium falciparum* and the presence or absence of malaria pigment granules. In positive smears, the number of asexual and sexual forms of *P. falciparum* in Giemsa-stained thick blood smears were counted against 1000 white blood cells assuming a normal count of 6000 leucocytes per microlitre of blood. A blood smear was declared negative if no parasite was found after examination of 100 microscope fields.

Calculation of parasite density for the positive films was by the formula below thus:

$${\text{Number of parasites}} \times 6000 \, = \, {{\text{Parasites}} \mathord{\left/ {\vphantom {{\text{Parasites}} {\upmu{\text{L}}}}} \right. \kern-0pt} {\upmu{\text{L}}}}$$

### Infant HIV diagnosis

All HIV exposed infants were tested for HIV using DNA PCR by collecting the dried blood sample (DBS) at the age of 6 weeks. The collected samples were sent to the Virology Department of Nnamdi Azikiwe University Teaching Hospital (NAUTH), Nnewi in Anambra State for DNA PCR testing.

### Disclosure of results

The disclosure of result was done after counselling. Infants with confirmed HIV patients were referred to the paediatric infectious disease unit of the hospital for further evaluation and management.

### Results validation and interpretation

HIV-1 DNA detected: when HIV-1 OD > 0.8 (regardless of IC OD).

HIV-1 DNA not detected: when HIV-1 OD < 0.2 and IC OD > 0.2

Invalid: when HIV-1 OD < 0.2 and IC OD < 0.2, needed retest.

Indeterminate: when HIV-1 OD was between 0.200 and 0.800, and/or if HIV-1 + sample was in a well next to a positive control or if a new HIV-1 + sample was next to a well with a previously known HIV + sample, needed retest.

### Statistical analysis

Statistical analysis of the result was done using SPSS version 20.0. There were cross tabulations to explore relationships between variables. The level of statistical significances was set at P-value 0.05 providing 95% confidence interval. Risk estimate was done to determine the relative risk of MTCT of HIV infection among women with placenta malaria. The Chi square test was used to determine associations between the categorical variables while linear logistic regression was used to determine the independent risk factors for placenta malaria.

The ethical approval for this study was obtained from the institutional ethical committee (Imo State University Teaching Hospital, Orlu). The study was carried out in accordance with the Code of Ethics of the World Medical Association (Declaration of Helsinki) for experiments involving humans. Informed consent was obtained from all the participants.

## Results

A total of 174 HIV positive pregnant women participated in this study. All the women received antiretroviral therapy. Seventy-eight (44.8%) had placenta malaria and 30 (17.2%) of the infants tested positive to HIV infection at 6 weeks. Table 1: shows the relationship between placenta malaria density and infant HIV status. There was significant relationship between placenta malaria density and infant HIV status (P-value < 0.001). The number of infants with HIV infection among women with maternal placental malarial parasitaemia was 30/78 (38.5%), while it was 0/96 (0%) for women without placenta malaria. Hundred percent (100%) of the infants whose placental malaria density is more than five thousand (> 5000) are HIV positive while 96% whose placental malaria density were between 0 and 5000 were HIV negative and 4%. The relative risk of developing HIV was 25% with confidence interval of 11.41–54.76% more in infants whose placental malaria were more than five thousand (> 5000) than in infants whose placental malaria density are between 0 and 5000. The relative risk of mother to child transmission of HIV was 25%. Table 2 shows the relative risk of MTCT of HIV infection.

**Table 1:** Placental malaria and infant HIV status

Placental malaria parasite density	Infant HIV status			
	Positive	Negative	Total	P-value = 0.001
0	0	96	96	
1–100	5	25	30	
101–1000	0	8	8	
1001–3000	0	14	14	
3001–5000	1	1	2	
Greater or equal to 5001	24	0	24	
Total	30	144	174

**Table 2:** Relative risk of MTCT of HIV in women with placental malaria

Placental malaria (copies/ml)	Infant HIV status		Total	χ^2^	P-value	RR	95% CI
	Negative	Positive		134	< 0.01	25%	11.42–54.76
> 5000	0 (0%)	24 (100%)	24 (100%)				
0–5000	144 (96%)	6 (4%)	150 (100%)				
Total	144 (82.8%)	30 (17.2%)	174 (100%)				

*RR* relative risk, *CI* confidence interval

The mean age of the women was 29.1 ± 4.2 year. Table 3 shows the association between social characteristics and placenta malaria density. There was no significant relationship between marital status and maternal placental malaria density (P-value + 0.13).

**Table 3:** Placental malaria and sociodemographic variables

Factors	Placental malaria (%)		*χ*^2^-value	*P*-value
	0–5000	> 5000		
Age range			2.79	0.59
< 20 years	1 (0.67)	0		
20–24	22 (14.67)	5 (20.83)		
25–29	56 (37.33)	5 (20.83)		
30–34	51 (34.0)	10 (41.67)		
35 years and above	20 (13.33)	4 (16.67)		
Educational status			6.50	0.09
None	3 (2.0)	1 (4.35)		
Primary	6 (4.0)	2 (8.7)		
Secondary	100 (66.67)	19 (82.61)		
Tertiary	41 (27.33)	1 (4.35)		
Marital status			7.20	0.13
Currently married	144 (96.0)	23 (95.83)		
Divorced	0	1 (4.17)		
Separated	1 (0.67)	0		
Single	4 (2.67)	0		
Widowed	1 (0.67)	0		

Table 4 shows selected obstetrics characteristics and placental malarial density. Early gestational age at booking was associated with lower level of maternal placental malaria (P-value < 0.01). Maternal placental malaria rate at booking gestational age < 14 weeks, > 14 weeks and up to 27 weeks and > 28 weeks were 47.4% (9 of 19) 45.5% (70 of 154) and 100% (1 of 1), respectively. There was an inverse relationship between CD4 count at booking and 36 weeks with the malaria placenta density with P-value of < 0.01 and 0.01, respectively.

**Table 4:** Placental malaria and selected obstetrics variables

Factors	Placental malaria (%)		*χ*^2^-value	*P*-value
	0–5000	> 5000		
Parity			1.37	0.50
Para 1	44 (29.33)	8 (33.33)		
Para 2–4	96 (64.0)	13 (54.17)		
Para 5 and above	10 (6.67)	3 (12.5)		
Gestational age			19.64	<0.01*
< 14 weeks	18 (12.16)	1 (4.17)		
14–20 weeks	121 (81.76)	15 (62.5)		
21–27 weeks	8 (5.41)	8 (33.33)		
28–35 weeks	1 (0.68)	0		
Booking CD4 count				
< 250	5 (3.33)	4 (16.67)	15.06	0.01*
250–350	16 (10.67)	7 (29.17)		
351–500	86 (57.33)	9 (37.5)		
> 500	43 (28.67)	4 (16.67)		
CD4 count at 36			13.20	0.01*
< 250	4 (2.67)	2 (8.33)		
250–350	13 (8.67)	7 (29.17)		
351–500	68 (45.33)	11 (45.83)		
> 500	65 (43.33)	4 (16.67)		

* Statistically significant

There was a significant relationship between the use of ITN, malaria treatment/prophylaxis and placenta malaria density. Pregnant women who slept under insecticide-treated net during the pregnancy period were less likely to have placenta malaria (P-value = 0.01). Women who received 3 doses of IPT during the period of antepartum period were less likely to have placenta malaria (P-value = 0.04). Women who were treated for symptomatic malaria during antepartum period were less likely to have placenta malaria (P-value < 0.01). Table 5 shows malaria prevention/treatment and placenta malaria density. The independent risk factors for placenta malaria include CD4 count at booking less than 350 and patients not treated for symptomatic malaria in pregnancy. This is shown in Table 6.

**Table 5:** Placental malaria and malaria preventive/treatment variables

Factors	Placental malaria (%)		*χ*^2^-value	*P*-value
	0–5000	> 5000		
Use of ITN			7.67	0.01*
Yes	120 (80.0)	13 (54.17)	7	
No	30 (20.0)	11 (45.83)		
IPT dose			8.39	0.04*
0	1 (0.67)	0		
1	6 (4.0)	1 (4.17)		
2	104 (69.33)	23 (95.83)		
3	39 (26.0)	0		
Treatment of malaria			144.30	< 0.01*
Yes	145 (97.32)	24 (100)	144.30	
No	4 (2.68)	0		

* Statistically significant

**Table 6:** Multiple linear regression model for predictors of placental malaria density

Independent significant factors	Coefficient	Standard error	*P*	95% Confidence interval	
				Upper	Lower
Gestational age (weeks)					
< 14	1	1	1	1	1
14–20	167.60	297.85	0.574	− 420.74	755.96
21–27	− 360.73	434.87	0.408	− 1219.73	498.26
28–35	− 329.80	1229.03	0.789	− 2757.49	2097.89
Booking CD4 count					
351–500	1	1	1	1	1
< 250	1639.69	712.70	0.023*	231.89	3047.49
250–350	1027.12	458.58	0.027*	121.29	1932.96
> 500	− 52.89	306.05	0.863	− 657.45	551.65
CD4 count at 36					
351–500	1	1	1	1	1
< 250	− 1143.2	792.62	0.151	− 2708.87	422.47
250–350	− 821.94	511.78	0.110	− 1832.87	188.98
> 500	− 308.30	291.46	0.292	− 884.04	267.42
Use of ITN					
Yes	1	1	1	1	1
No	110.78	224.92	0.623	− 333.51	555.07
IPT dose					
3 doses	1	1	1	1	1
0 dose	281.07	1216.53	0.818	− 2121.92	2684.07
1 dose	− 927.64	506.84	0.069	− 1928.80	73.51
2 doses	517.28	239.10	0.032*	44.98	989.58
Treatment of malaria					
Yes	1	1	1	1	1
No	6357.33	283.13	< 0.001*	5798.06	6916.60

* Statistically significant

## Discussion

The overall rate of mother-to-child transmission (MTCT) of HIV infection of 17.2% and placental malaria parasitaemia rate of 44.8% were recorded in this study. The rate of MTCT among women with placenta malaria was 38.5%. The rate of MTCT and prevalence of placenta malaria in this study are comparable to rates reported from some studies in other sub-Saharan African countries [28–32]. Ayisi et al. [20], recorded an overall rate of MTCT of HIV infection of 19.9% and 21.9% among women with placenta malaria which is lower than 38.5% in our study. However, Ayisi excluded women with low CD4 count and sick HIV patients which could have accounted for the observed difference. The placental malaria parasitaemia prevalence of 44.8% in this study is high compared to 25% to 26.4% in the studies done in Minna Nigeria, Burkina Faso and Gambia [33–36] but lower compared to 63.9% in a study in South East Nigeria by Umeh et al. [37]. The lower prevalence in Burkina Faso could be linked to the low prevalence of HIV infection in the country, This disparity in values in other part of Nigeria may be due to difference in endemicity and seasons at which the studies were conducted [38]. It was observed that placental malaria parasitaemia was significantly associated with MTCT of HIV. This result is consistent with some studies conducted in Uganda [29], Kenya [22], Malawi [30], and Tanzania [32].

Most patients who had symptomatic malaria and were treated with artemisinin-based combination therapy had low placental malaria parasitaemia and low rate of MTCT of HIV when compared with patients who had less than two doses of IPT for malaria with sulfadoxine-pyrimethamine. There is urgent need to explore the role of artemisinin-based combination therapy (ACT) in reducing the placenta malaria and rate of MTCT of HIV infection. The use of anti-malarial medication is known to reduce HIV-1 replication and viral loads in adult [38, 39].

This study showed a significant difference in the rate of MTCT of HIV among the users of ITN and no users. This corroborates the use of ITN in all trimesters and all times advocated by the World Health Organization as one of the strategies for reduction of malarial burden in pregnancy [40]. This is consistent with a Cochrane review [41] which showed that in Africa, use of ITN compared with no nets significantly reduced placental parasitaemia. Though malaria prevention measures awareness was high in this study (98.8%), the percentage use of ITN was 76.4%. This may account for the high placental malarial parasitaemia in this study despite wide spread use of IPT for malaria.

The predictors of placental malarial parasitaemia and eventual MTCT of HIV infection in this study included, gestational age at booking, CD4 counts, treatment of symptomatic malaria with ACT and number of doses of IPT for malaria which are comparable to other studies [22, 41]. However, the use of IPT was not an independent factor for placenta malaria. This calls for further studies and possible review of the strategies for malaria prevention among pregnant women with HIV infection. The high placental malaria parasitaemia prevalence and MTCT of HIV infection recorded in this study is unacceptable from the background of huge human and financial resources deployed into PMTCT of HIV.

One of the strengths of this study was that it evaluated the association between the placenta malaria density and MTCT of HIV infections. It was observed that women whose placenta has a malaria density greater than 5000 were 2.5 times more likely to transmit the virus to their offspring. The study also determined the effects of the prophylactic and therapeutic malaria drugs on placenta malaria density and MTCT transmission of HIV infection.

Despite the obvious strengths, the result should be interpreted with caution because it is a single centre study and cross-sectional study. Another limitation of this study was that it did not estimate the viral load, which has been shown to increase when the placenta malaria parasite density is very high. However, it documented the CD4 count which is used to monitor patients in many centres in developing countries. The study showed an inverse relationship between the CD4 count and the malaria parasite placenta density. This is consistent with the observation that malaria parasite affects the helper T-cells. The inability to assess the nutritional status was also another limitation of the study.

## Conclusion

In view of the findings from this study, there is need to determine the resistance pattern of sulfadoxine-pyrimethamine in this environment and to explore other alternatives for malaria prophylaxis in HIV positive pregnant women. It is hypothesized that ACT may be beneficial in reducing density of placenta malaria and MTCT of HIV infections in sub-Saharan Africa. More studies will be needed to establish the beneficial role of artemisinin-based combinations as more effective drugs for IPT in reducing placental parasitaemia compared with currently used sulfadoxine-pyrimethamine for IPT. Optimizing the conditions of these women before pregnancy by reducing the viral load and increasing the CD4 count should be a potential target for reducing placenta malaria and mother to child transmission of HIV infection.

## Data Availability

The datasets used and/or analysed during the current study are available from the corresponding author on reasonable request.

## Abbreviations

ACT: Artemisinin-based combination therapy
AIDS: Acquired immunodeficiency syndrome
CD4: cluster of differentiation 4
DBS: Dried blood sample
DNA: Deoxy ribonucleic acid
HIV: Immunodeficiency virus
IPT: Intermittent preventive treatment for malaria
ITN: Insecticide-treated net
HAART: Highly active anti-retroviral therapy
MTCT: Mother to child transmission
NAUTH: Nnamdi Azikiwe University Teaching Hospital
PCR: Polymerase chain reaction
PMTCT: Mother to child transmission of HIV infection
OD: Optical density
SPSS: Statistical package for social science
